# The complete mitochondrial genome of a Buckfast bee, *Apis mellifera* (Insecta: Hymenoptera: Apidae) in Northern Ireland

**DOI:** 10.1080/23802359.2018.1450660

**Published:** 2018-03-13

**Authors:** Hisashi Okuyama, John Hill, Stephen John Martin, Jun-ichi Takahashi

**Affiliations:** a Faculty of Life Sciences, Kyoto Sangyo University, Kyoto, Japan;; b Randalstown and District Beekeepers’ Association, Crumlin Co Antrim, UK;; c School of Environment and Life Sciences, University of Salford, Manchester, UK

**Keywords:** Buckfast bee, origin, Ireland, *Apis mellifera ligustica*, honeybee

## Abstract

We analyzed the complete mitochondrial genome of the ‘Buckfast bee’, *Apis mellifera,* collected from North Ireland, UK. It consisted of a circular molecule of 16,353 bp. The genome contained 13 protein-coding, 22 tRNA, and 2 rRNA genes, along with one A + T-rich control region. The average AT content was 84.9%. The genes ATP8 and ATP6 shared 19 nucleotides. A phylogenetic analysis, suggested that the matriline ‘Buckfast bee’ has remained most closely related to the *A. mellifera ligustica* race from which it originated in 1917, despite being cross-bred with many other *A. mellifera* races over the past 100 years.

The Buckfast bee, *Apis mellifera,* was developed by Brother Adam at Buckfast Abbey, in South-West England, originally for their resistance towards the acarine mite, calmness on the comb and excellent honey production, all properties beekeepers value in *A. mellifera* (Adam 1987; Olszewski 2009; Holm 2014). In 1917, Brother Adam started breeding his new bee ‘Buckfast’ line with a few Italian queens (*A. mellifera ligustica*) (Adam 1987; Holm 2014). Over the following decades, he continually selected for traits he favored by crossing his ‘Buckfast line’ with a wide variety of bee races from across Europe (*A. m. mellifera, A. m. ligustica,* and *A. m. cecropia*), near Asia (*A. m. anatoliaca*), and Africa (*A. m. sahariensis* and *A. m. monticola*). Since his death in 1996, Buckfast Abbey and several honeybee breeder groups across Europe have continued to maintain the Buckfast strain on islands and other isolated places (Adam 1987; Holm 2014). Due to the large amount of cross-breeding with many different races, the ancestral lineage of the Buckfast bee remains unclear. Here, we analyzed the complete mitochondrial genome in order to help determine the maternal origin of the current ‘Buckfast bees’ maintained in Northern Island, UK.

An adult worker of a Buckfast bee queen previously crossed with an *A. m. cecropia* male in an apiary of Northern Ireland was collected in March 2017 (The specimen was stored in the National Museum of Nature and Science, Japan, accession number: NSMT-I-HYM 75326). Genomic DNA was extracted from its thoracic muscle tissue using a standard phenol/chloroform method. This was then sequenced using Illumina's MiSeq platform (Illumina, San Diego, CA). The resultant reads were assembled and annotated using the MITOS web server (Bernt et al. 2013) and Geneious R9 (Biomatters, Auckland, New Zealand). The phylogenetic tree was constructed using MEGA6 (Tamura et al. 2013) and TREEFINDER (Jobb 2015) using the nucleotide sequences of the 13 protein-coding genes (PCGs).

The mitochondrial genome of the ‘Buckfast bee’ consisted of a closed loop containing 16,353 bp (AP018432). That contained a heavy (H)-strand, which encoded nine PCGs and 14 tRNA genes, whereas the light (L)-strand encoded four PCGs, eight tRNA, and two rRNA genes. The start codon was ATT for the six PCGs; ATG for four PCGs; ATA for *COI* and *ND3*, and ATC for *ND2*. The stop codon for all PCGs was TAA.

The *A. mellifera* haplotype of the non-coding region between tRNA-*Leu* and *COII* genes was the C lineage sequence, which was consistent with the findings of previous studies based on *A. m. ligustica* (Cornuet and Garnery 1991; Franck et al. 2000; Dall’Olio et al. 2007). A phylogenetic analysis was constructed using 13 mitochondrial PCGs across 17 *Apis* species (Figure 1). This phylogenetic analysis suggested that the maternal line of the ‘Buckfast bee’ in Northern Ireland is most closely related to *A. m. ligustica* reflecting to persistence of the original Italian matrilines from which the Buckfast bee was originally derived from in 1917, over 100 years ago (Adam 1987; Holm 2014). The genetic distance between the ‘Buckfast bee’ and *A. m. ligustica* mitochondrial genome was 0.00036, which corresponds well to the genetic distance generally observed within *A. mellifera* subspecies.

**Figure 1.**
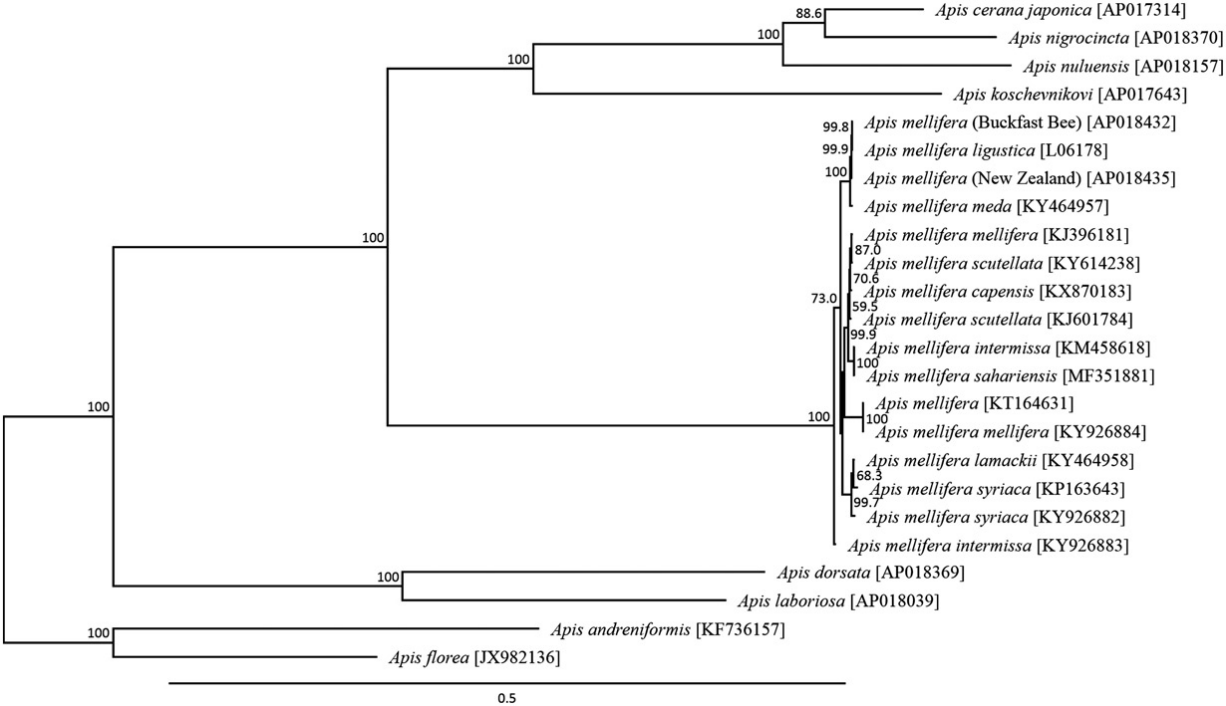
Phylogenetic relationships (maximum likelihood) of the honey bees (*Apis* spp) and *Apis mellifera* races based on the nucleotide sequence of 13 protein-coding genes of the mitochondrial genome (Crozier and Crozier 1993; Gibson and Hunt 2015; Hu et al. 2015; Haddad 2015; Eimanifar et al. 2016a, 2016b; Eimanifar et al. 2017a, 2017b, 2017c; Haddad et al. 2017; Nakagawa et al. 2017). The numbers at the nodes indicate bootstrap support inferred from 1000 bootstrap replicates. Alphanumeric terms indicate the GenBank accession numbers.
